# Clinical Evaluation of Acetaminophen–Galgeuntang Interaction Based on Population Approaches

**DOI:** 10.3390/pharmaceutics12121182

**Published:** 2020-12-04

**Authors:** Quyen Thi Tran, So Jung Park, Hyun-moon Back, Lien Thi Ngo, Duc Tuan Cao, Hung Van Nguyen, Sangkeun Jung, Jung-woo Chae, Yang Chun Park, Hwi-yeol Yun

**Affiliations:** 1 College of Pharmacy, Chungnam National University, Daejeon 34134, Korea; quyentran@cnu.ac.kr (Q.T.T.); lienngovn@cnu.ac.kr (L.T.N.); 2 Daejeon Korean Hospital of Daejeon University, Daejeon 35235, Korea; vivies@hanmail.net; 3 Department of Pharmaceutics, Ernest Mario School of Pharmacy, Rutgers, The State University of New Jersey, Piscataway, NJ 08854, USA; hmback89@gmail.com; 4 Department of Pharmaceutical Chemistry and Quality Control, Faculty of Pharmacy, Haiphong University Medicine and Pharmacy, Haiphong 180000, Vietnam; cdtuan@hpmu.edu.vn; 5 Department of Pharmacology, Faculty of Pharmacy, Haiphong University Medicine and Pharmacy, Haiphong 180000, Vietnam; nvhung@hpmu.edu.vn; 6 Department of Computer Science and Engineering, Chungnam National University, Daejeon 34134, Korea; hugmanskj@gmail.com

**Keywords:** acetaminophen, Galgeuntang, population pharmacokinetics, drug interaction

## Abstract

Galgeuntang (GGT), a traditional herbal medicine, is widely co-administered with acetaminophen (AAP) for treatment of the common cold, but this combination has not been the subject of investigation. Therefore, we investigated the herb–drug interaction between GGT and AAP by population pharmacokinetics (PKs) modeling and simulation studies. To quantify PK parameters and identify drug interactions, an open label, three-treatment, three-period, one-sequence (AAP alone, GGT alone, and AAP and GGT in combination) clinical trial involving 12 male healthy volunteers was conducted. Ephedrine (EPD), the only GGT component detected, was identified using a one-compartment model. The PKs of AAP were described well by a one-compartment model and exhibited two-phase absorption (rapid followed by slow) and first-order elimination. The model showed that EPD significantly influenced the PKs of AAP. The simulation results showed that at an AAP dose of 1000 mg × 4 times daily, the area under the concentration versus time curve of AAP increased by 16.4% in the presence of GGT compared to AAP only. In conclusion, the PKs of AAP were affected by co-administration of GGT. Therefore, when AAP is combined with GGT, adverse effects related to overdose of AAP could be induced possibly.

## 1. Introduction

Herbal medicines are widely used alone or in combination with synthetic drugs. Co-administration of herbal medicines and synthetic drugs may result in drug interactions, which could affect efficacy of treatment [1]. Indeed, a large number of over-the-counter combinations of synthetic and herbal drugs is used to treat mild conditions, despite the dearth of data on their interactions.

Galgeuntang (GGT), a traditional herbal medicine, is extracted from the herbs *Puerariae Radix*, *Ephedra sinica*, *Paeonia albiflora*, *Glycyrrhiza uralensis Fisch*, *Zingiber officinale Roscoe*, *Cinnamomum cassia*, and *Ziziphus jujuba*. According to Korean Herbal Pharmacopoeia (2013), puerarin, ephedrine, paeoniflorin, glycyrrhetic acid, and (6)-shogaol are confirmed as marker compounds of GGT. As a result of its anti-inflammatory, anti-pyretic, analgesic [2], antioxidant [3], and anti-influenza virus [4] activities, GGT has been used to treat the common cold. In addition, GGT has been reported to modulate atopic dermatitis [5] and chronic rhinosinusitis [6]. GGT is frequently co-administered with Tylenol (acetaminophen) to treat the common cold in East Asia. Although regulatory agencies approved both drugs based on their efficacy and safety, no clinical study of this combination has been conducted.

Herbal medicines contain a variety of components, making them susceptible to interactions with other drugs. Such interactions can alter the pharmacokinetics (absorption, distribution, metabolism, and excretion) of co-administered drugs. GGT with five major ingredients also possesses chance of drug interactions when co-administering with other drugs. A number of in vitro studies investigated the influence of GGT and GGT components on cytochrome (CYP) 450 and predicted its drug interactions [7,8,9,10,11,12,13,14,15,16,17,18,19]. For example, GGT reportedly inhibits CYP2C19, CYP2D6, and CYP2E1 [16]. Glycyrrhetic acid completely inhibits CYP3A4 [12], and paeoniflorin inhibits the activity of CYP1A2 in vivo [17]. Wu et al. [8] reported that ephedrine induces CYP1A2 activity. Moreover, puerarin inactivates CYP2C19, CYP2D6, and CYP1A2 [10].

Acetaminophen (AAP) metabolism mainly occurs in the liver and a lesser extent in the kidney and intestine [20]. AAP is primarily metabolized to AAP glucuronide and AAP sulfate, and with a minor fraction to *N*-acetyl-*p*-benzoquinone imine (NAPQI) [21]. NAPQI is highly reactive and mediates AAP-related hepatotoxicity. Several CYP450 (CYP3A4, CYP1A2, CYP2D6, CYP2A6, and CYP2E1) were reported to convert AAP to NAPQI [21]. Therefore, when GGT and AAP are co-administered, a potential herb–drug interaction might occur and be mediated by CYP450. It possibly results in increasing NAPQI level and consequently causes hepatotoxicity. However, no clinical study has been performed to evaluate such interaction to date. Therefore, our study was conducted to investigate the effect of GGT on PKs of AAP by performing population pharmacokinetics and simulation studies.

## 2. Materials and Methods

### 2.1. Materials

GGT tablets (640 mg) were purchased from Kyungbang Pharmaceutical Company (394 Namdong-daero, Namdong-gu, Incheon, Republic of Korea). The solvent used in the extract was 100% H_2_O and the ratio of herbal drug to extract were 2.78 to 1 (*Puerariae Radix*), 4.35 to 1 (*Ephedra sinica*), 3.03 to 1 (*Paeonia albiflora*), 2.27 to 1 (*Glycyrrhiza uralensis Fisch*), 5.58 to 1 (*Zingiber officinale Roscoe*), 1.96 to 1 (*Ziziphus jujuba*), and 9.09 to 1 (*Cinnamomum cassia*). Major ingredients in one tablet GGT were determined by high-performance liquid chromatography-tandem mass spectrometer (HPLC-MS/MS) method and the content of each ingredient are listed in Table 1. Tylenol (AAP) tablet 500 mg was provided by Jansen Korea Ltd. (45, Jeyakgongdan 2-gil, Hyangnam-eup, Hwaseong-si, Gyeonggi-do, Republic of Korea).

### 2.2. Participants

Twelve male healthy volunteers who met the inclusion and exclusion criteria participated in this study. The inclusion and exclusion criteria were provided in the Supplementary Material Table S1. All participants gave their informed consent before participating the study. The study was performed in accordance with the Declaration of Helsinki, and the protocol was approved by the Ethics Committee of Daejeon Korean Hospital of Daejeon University (Project identification no. DCDC_18_01, approved on 3 January 2018).

### 2.3. Clinical Trial

This open-label, three-treatment, three-period, one-sequence clinical trial involved 12 male healthy volunteers. Each participant received three treatments with a 7 ± 1-day washout period in-between. The treatment schedule was as follows: period 1, (G1) single-dose AAP 500 mg (2 tablets); period 2, (G2) combination of AAP 500 mg (2 tablets) and GGT 640 mg (12 tablets); and period 3, (G3) single-dose GGT 640 mg (12 tablets). A dose of 1000 mg AAP (equal to 2 tablets) was used in this study because in clinical practice, AAP is recommended at 1000 mg for the treatment of the common cold. To enhance efficacy of AAP, GGT is suggested in combination with AAP, and the maximum recommended dose of GGT is 12 tablets per day. Therefore, we applied this dose of GGT in our study.

**Figure 1:**
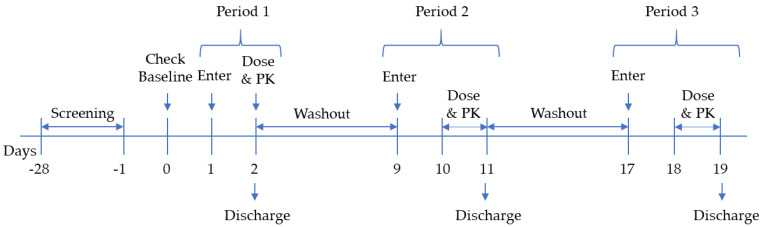
Scheme of the clinical trial. PK, pharmacokinetics.

After screening (days −28 to −1), participants underwent a baseline assessment (day 0). All participants fasted from 10 p.m. on the night prior to the day of drug administration. During period 1, participants were given the drug at 8 a.m. and resided in the study center for at least 7 h for sample collection. In periods 2 and 3, the subjects stayed in the center for at least 30 h for PK analysis. The study design is shown in Figure 1.

### 2.4. Drug Quantification

Blood samples were analyzed by high-performance liquid chromatography-tandem mass spectrometry (Agilent 1100 HPLC; AB Science API2000). Analytes were detected by multiple reaction monitoring at 152.2 → 110.1, 471.2 → 135.2, 277.2 → 137, 503.2 → 341.2, 166.2 → 117.1, 417 → 267.1, and 426.3 → 175.1 (precursor → product *m/z*) for AAP, glycyrrhetic acid, (6)-shogaol, paeoniflorin, EPD, puerarin, and domperidone (internal standard), respectively. The calibration curves for AAP and EPD were linear at 50 to 10,000 ng/mL and 50 to 2500 ng/mL, respectively (R^2^ > 0.99). If concentrations of AAP or EPD were measured outside of linear range, they would be diluted and re-measured to fit the calibration curves. The intra-day and inter-day accuracies and precisions of AAP and EPD was provided in Supplementary Materials Table S2. The analytical methods for (6)-shogaol and puerarin were developed with linear range of 100 ~ 2500 ng/mL and 50 ~ 2500ng/mL, respectively. However, both of components in GGT were detected under Limit of quantitation in whole plasma samples. Two remaining components, glycyrrhetic acid and paeoniflorin, were not detected in our plasma samples. The analytical column was a Gemini-C18 (5 µm, 50 × 4.6 mm). The compounds were separated using a stationary mobile phase consisting of 0.5% formic acid and methanol at a ratio of 20:80 *v/v* and a flow rate of 0.4 mL/min. Representative chromatograms of blank plasma, blank plasma sample spiked with internal standard, standard samples of AAP and EPD, and a real plasma sample of AAP and EPD were provided in Supplementary Materials Figure S2.

### 2.5. Non-Compartmental PK Analysis

Non-compartmental PK analysis was performed using Phoenix WinNonlin (version 8.2.0.4383, Certera LP). The PK parameters were compared between the two groups by Mann–Whitney U-test (IBM SPSS Statistics Version 24). The level of statistical significance was set at α = 0.05 for all tests. The area under the concentration–time profile curve from zero to the last time point (*AUC*_0-t_) was calculated by the linear log trapezoidal method. Terminal rate constant (*λ_z_*) was calculated by performing a set of linear regressions of the log(concentration)–time data using at least three sample points from the terminal portion of the curve. Terminal elimination half-life (*t*_1/2_) was calculated as *t*_1/2_ = ln(2)/*λ_z_*. The *AUC* from zero to infinity (*AUC*_inf_) was derived as total *AUC* extrapolated to infinity using the terminal rate constant, *AUC*_inf_ = *AUC*_0-t_ + *C*_last_/*λ_z_*, where *C*_last_ is the last observed concentration. *CL/F* was calculated as dose ÷ *AUC*_inf_. The *V/F* was calculated as *CL/F/λ_z_*, where *F* is bioavailability.

### 2.6. Population PK Analysis

A population PK analysis was conducted by nonlinear mixed-effects modeling (NONMEM, version 7.4.1). PK parameters and interindividual and residual variabilities were estimated using first order conditional estimation with interaction. The model was modified in a sequential manner to identify that which best fit the data. The model was chosen based on the OFV and plots of goodness of fit. IIV was described using an exponential error model (Equation (1)):

*P*_i_ = *P*_pop_ × exp(*η*_i_) (1)

where *P*_i_ represents estimate value of parameter *P* for i*th* individual, *P*_pop_ represents typical value of parameter *P* at the population level, and *η*_i_ is the IIV which describes the difference between individual and population value. *η*_i_ was assumed to be normally distributed with mean of 0 and variance of ω^2^. Additive, proportional, and combination of additive and proportional error models were explored for residual variability and the proportional error model was chosen as the final residual variability model, since the other models were statistically insignificant (objective function value (OFV) increased 99.3 for additive model and combined-error model showed same OFV even though one more degree of freedom in comparison with proportional error model). Therefore, proportional error model was adopted in our study to describe the difference between observed and predicted values as below (Equation (2)):

*C*_ij_ = *C* × (1 + *ɛ*_pij_) (2)

where *C*_ij_ and *C* are the jth measured observation and prediction for the ith subject, respectively; *ɛ*_pij_ is the proportional random error which was assumed to have normal distribution with a mean of zero and a variance of σ^2^.

After development of optimal AAP model, a covariate analysis was conducted to assess additional variables as determinants of the variability in the PK estimate. The co-administered drug (GGT) was considered a covariate. Covariate was tested using forward selection (*p* < 0.05) and backward elimination (*p* < 0.01) processes with likelihood ratio test to determine whether GGT affects the PKs of AAP. The effect of GGT on PKs of AAP was tested by linear, exponential, and power function models. The differences between OFV of each model and the base model were calculated. During forward selection, a covariate was selected if it showed at least a decrease of 3.84 in OFV. In backward elimination, an increase of at least 6.64 unit of OFV was set as criteria for retaining a covariate.

The final model was validated by VPC and nonparametric bootstrapping. The parameter estimates of the bootstrap samples were re-estimated and the median and 95% confidence intervals were compared with those estimated using the final model. For VPCs, the estimated parameter and the final model were used to simulate 1000 data sets, and the observed data were overlaid on the simulated median and 90% prediction intervals.

### 2.7. Simulation Scenarios

To mimic the recommended dose based on the status of patients, AAP dose is suggested from 1000 mg to the maximum dose of 1000 mg × 4 times (8 tablets) per day for treatment of the common cold [22,23]. Twelve tablets of GGT 640 mg per day is the maximum recommended dose. Therefore, two simulation scenarios were used to evaluate the magnitude of any interaction, (AAP 1000 mg once daily + GGT 7680 mg once daily) and (AAP 1000 mg × 4 times daily + GGT 7680 mg once daily) in a week. The effect of GGT on the PKs of AAP was assessed by monitoring the *AUC*_t_, *C*_max_, and *t*_1/2_ of AAP in the presence and absence of GGT. The median plasma concentration of AAP over time was plotted. The *AUC*_t_ of one dose on day 7 was estimated using the linear log trapezoidal method. The magnitude of the influence of GGT on AAP was evaluated by calculating the ratio of *AUC*_t_ of AAP when co-administered GGT to *AUC*_t_ of AAP administered alone on day 7.

## 3. Results

### 3.1. Non-Compartmental PK Analysis

Among the components of GGT, only EPD (*Ephedra sinica*) was detected under our analysis. Therefore, we investigated the interaction between AAP and EPD.

**Figure 2:**
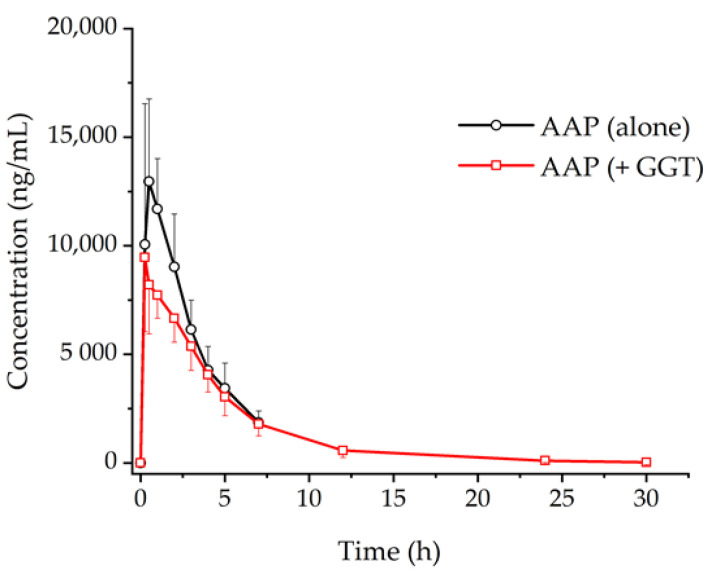
Comparison of plasma concentration–time profiles of acetaminophen (AAP) after administration alone or co-administration with Galgeuntang (GGT). Each point represents the mean and standard deviation.

Figure 2 shows the mean plasma concentration versus (vs.) time profiles of AAP administered alone (group 1, G1) and AAP co-administered with GGT (group 2, G2). Table 2 summaries PK parameters of AAP in the presence or absence of GGT according to non-compartmental analysis (NCA) results. The area under the concentration–time curve from time zero to infinity (*AUC*_inf_), the maximum plasma concentration (*C*_max_), the half-life (*t*_1/2_), and volume of distribution (*V/F*) of AAP significantly decreased in G2 in comparison to those in G1 (*p* < 0.001).

### 3.2. Population PK Analysis

#### 3.2.1. Base Model

One- and two-compartment with first-order elimination and absorption were initially tested in development AAP model. The objective function values (OFV) of those models are listed in Table 3. One-compartment model gave an OFV of 1493. When two-compartment was applied, the OFV increased by 34.6 units comparing to one-compartment model. Therefore, one-compartment was used in the next step.

**Figure 3:**
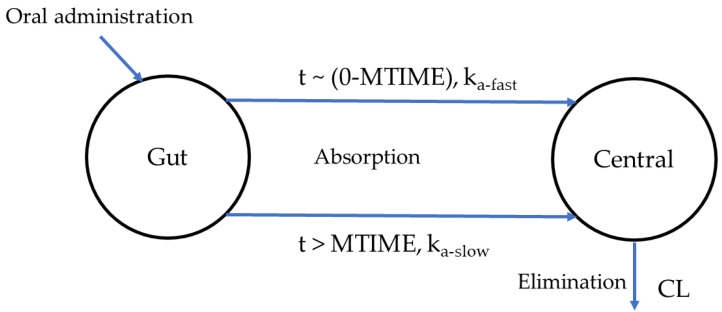
Scheme of the base model with two periods of absorption phase. CL, clearance.

Variety of absorption models was tested to describe absorption process of AAP. Gastric emptying (GE) was firstly modelled in absorption phase of AAP owing to the dependence of AAP absorption on rate of GE [24]. Another simple GE model was also taken into consideration due to sparse absorption data. In which, absorption phase was modelled with two periods, initially rapid absorbed (represented by *k_a__-fast_*) and followed by absorbed slowly (represented by *k_a__-slow_*). Results show that the greatest decrease of OFV was shown in the model with two periods of absorption phase. Therefore, this model was used in the covariate step and was considered the base model. Scheme of the base model is provided in Figure 3.

#### 3.2.2. Covariate Model and Drug Interactions

In the covariate analysis, the effect of co-administration EPD on the principal PK parameters (apparent clearance (*CL/F*), absorption rate (*k_a__-slow_* and *k_a__-fast_*), and volume of distribution (*V/F*)) of AAP was investigated. The criteria for selecting the final model are listed in Table 4. EPD was identified as a significant covariate for *V/F*, *k_a__-slow_*, and *k_a__-fast_* of AAP. Linear equations were used to describe the effect of EPD on PKs of AAP as below (Equations (3)–(5)):

*V*/*F* = *TVV* × (1 + *COV*_*V/F*_ × *DOSE*_*EPD*_) (3)

*K*_*a-fast*_ = *TVK*_*a-fast*_ × (1 + *COV*_*ka-fast*_ × *DOSE*_*EPD*_) (4)

*K*_*a*__*-slow*_ = *TVK*_*a*__*-slow*_ × (1 − *COV*_*ka*__*-slow*_ × *DOSE*_*EPD*_) (5)

where *TVV*, *TVK_a__-fast_*, and *TVK_a__-slow_* are the typical value of *V/F*, *k_a__-fast_*, and *k_a__-slow_* of AAP in the population without concomitant, respectively. COV*_V/F_*, COV*_Ka__-fast_*, and COV*_Ka__-slow_*represent the covariate on *V/F*, *k_a__-fast_*, and *k_a__-slow_* of AAP, respectively.

**Figure 4:**
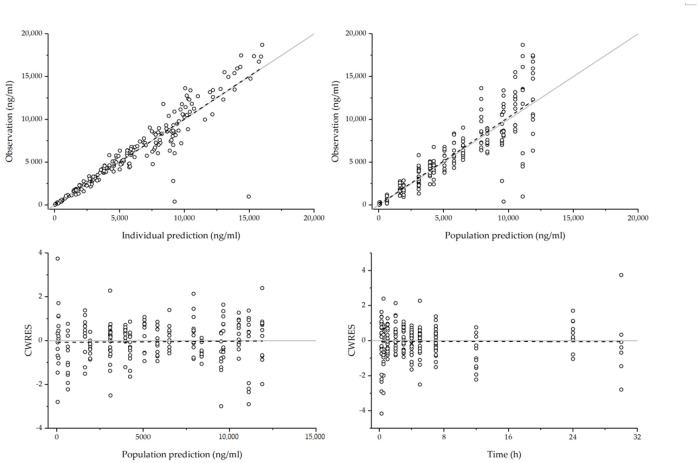
Goodness of fit plots of the final AAP model. Empty circle: observation, solid line: reference line (y = x line for individual predicted concentrations (IPRED) or predicted concentrations (PRED) vs. observation plots, y = 0 for Time or IPRED vs. conditional weight residual (CWRES) plots), dash line: regression line.

Incorporation of EPD significantly reduced the interindividual variability (IIV) and OFV (*p* < 0.05). IIV of *V/F* and *k_a__-slow_* was expressed as coefficient of variation, decreased from 16.9 to 0% and 22.9 to 0%, respectively. The final estimated parameters are listed in Table 5, and goodness of fit plots for the final AAP model are shown in Figure 4. Plots of population predicted concentrations (PRED) vs. observed concentration and individual predicted concentrations (IPRED) vs. observed concentration showed a symmetric distribution. In addition, in plots of conditional weight residual (CWRES) vs. PRED and CWRES vs. time, the plasma concentration showed a symmetry about CWRES = 0. Therefore, the model adequately described the observed plasma concentration.

#### 3.2.3. Model Evaluation

**Figure 5:**
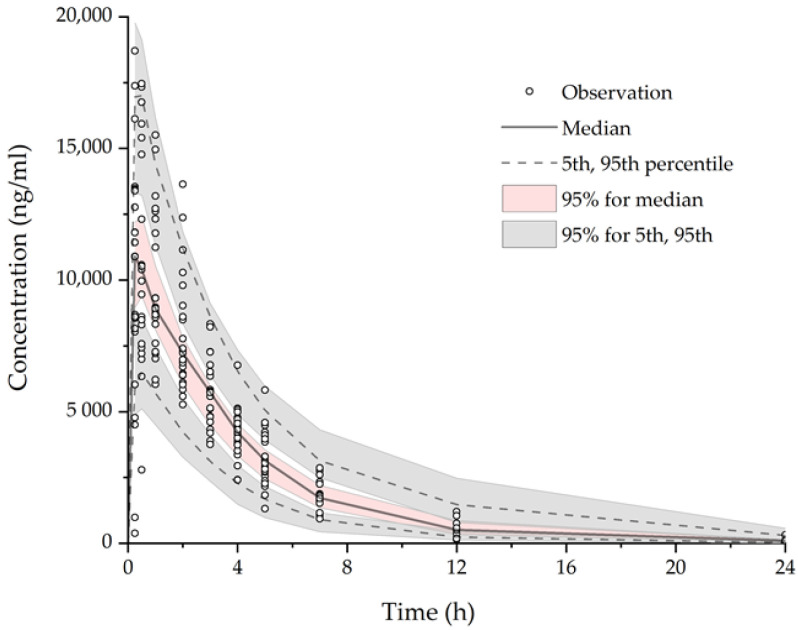
Visual predictive check (VPC) plot of the final model for AAP observations. Empty circles: observed AAP concentration; pink area: 95% confidence intervals of median predicted concentration; gray areas: 95% confidence intervals of 5th and 95th percentile predicted concentration.

The parameter estimates and 95% confidence intervals for the parameters were calculated (Table 5). All estimates obtained from the final model were comparable to the bootstrap estimates (*n* = 1000) and were within the 95% bootstrap confidence intervals. Figure 5 shows the result of visual predictive check (VPC) (*n* = 1000) for AAP. Overall, the VPC suggested that the final model adequately describes the distribution of AAP concentration. The simulated median represented the trend of the observed concentration. In addition, the majority of the observed values fell within the 90% prediction intervals.

### 3.3. Simulation of Drug Interaction

Simulations were performed 1000 times using the developed model to assess the effect of GGT on PKs of AAP. AAP at 1000 once a day or 1000 mg × 4 times a day was administered in the presence or absence of GGT (7680 mg) daily. The drugs were given consecutively over 1 week. The result was provided in the Table 6.

**Figure 6:**
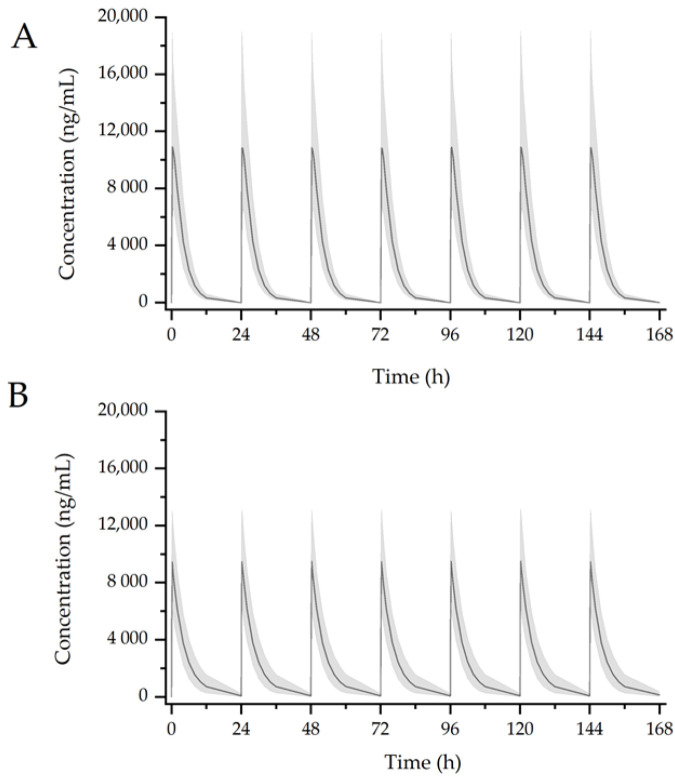
Simulation result of 1000 mg AAP administered alone (**A**) or co-administered with GGT (**B**) in a week. Black solid lines: median simulated concentration; shaded bands: 90% percent confidence interval for 1000 replicates simulation.

**Figure 7:**
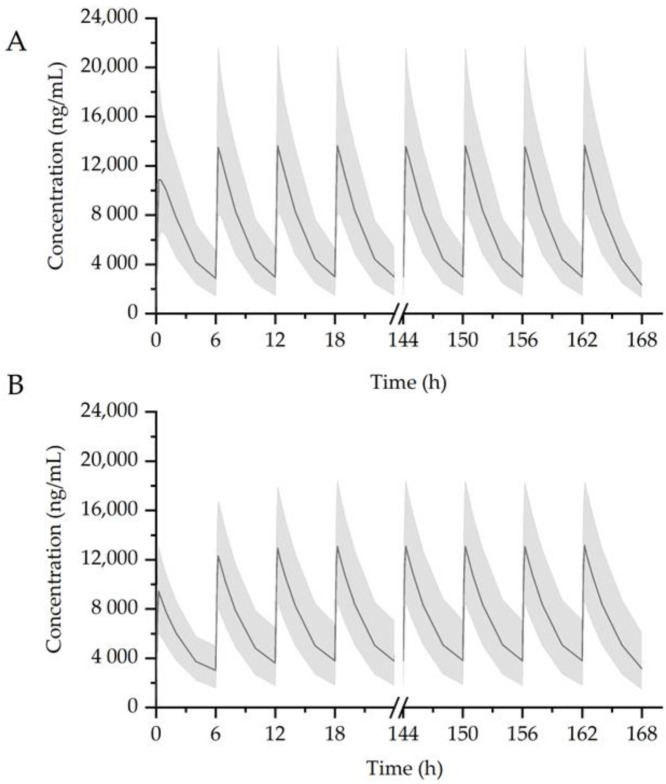
Simulation result of 1000 mg × 4 times per day of AAP administered alone (**A**) or co-administered with GGT (**B**) in a week. Black solid lines: median simulated concentration; shaded bands: 90% percent confidence interval for 1000 replicates simulation.

The median predicted AAP plasma concentrations are shown in Figure 6 and Figure 7. AAP administered alone (1000 mg; Figure 6A) resulted in the following PK parameters: *AUC*_t_ = 43,606 ng/mL h, *C*_max_ = 10,872 ng/mL, and *t*_1/2_ = 2.17 h. In the presence of GGT (Figure 6B), the steady-state PK parameters of AAP were as follows: *AUC*_t_ = 43,024 ng/mL h, *C*_max_ = 9474 ng/mL, and *t*_1/2_ = 4.69 h. The *AUC* of AAP in combination with GGT is comparable to that in group of AAP administered alone, which was unlikely to be clinically significant. Simulation of 1000 mg × 4 times per day of AAP administered alone yielded the following steady state PK parameters: *AUC*_t_ = 40,940 ng/mL h, *C*_max_ = 13,679 ng/mL, and *t*_1/2_ = 2.17 h (Figure 7A). When co-administered with GGT, the *AUC*_t_ and *t*_1/2_ increased to 42,854 ng/mL h and 2.8 h, respectively (Figure 7B), while the predicted *C*_max_ decreased to 13,149 ng/mL, comparing to AAP administered alone.

## 4. Discussion

We investigated the effect of GGT on PKs of AAP in 12 healthy males using a population PK modeling approach. The PKs of AAP were best described by a one-compartment and first-order elimination and two-period absorption phases. EPD, the only component of GGT detected, was identified as a covariate in the model. The *CL/F*, *V/F*, *k_a__-fast_*, and *k_a__-slow_* of AAP were estimated to be 22.5 L/h, 16.3 L, 0.970 L/h, and 0.320 L/h when administered alone, and 22.5 L/h, 84.6 L, 7.60 L/h, and 0.0901 L/h when administered with GGT, respectively.

In our study, GE was applied to describe absorption process of AAP due to the dependence of AAP absorption on the rate of GE [24]. A biphasic GE pattern was modelled, in which a fraction of total administered drug (*f_1_*) rapidly reaches the systemic circulation (SC) in a short time (around 10–15 min), and later, the remaining fraction (*f_2_*) releases from the stomach to the small intestine and enters SC followed by mono-exponential model [25]. Therefore, we described the pattern in the model using MTIME options to make it simple (two periods of absorption phase). In which, *f_1_* is quickly absorbed into SC with rapid absorption rate (*k_a__-fast_*), and after the amount of time (MTIME), *f_2_* enters SC with lower absorption rate (*k_a__-slow_*). This model was confirmed to well describe absorption process of AAP than the others (e.g., transit absorption mode, lag time) in our study.

Since the dependence of AAP absorption on GE rate [24], absorption of AAP is susceptible to factors which can change GE rate, such as food or concomitant intake. In this study, for GGT 7680 mg, a total of 12 tablets were co-administered with AAP. Those of tablet intake was enough to stimulate gastro-intestinal (GI) mobility under fasting condition, so the *k_a__-fast_* of AAP could be increased; however, it was not a clinically meaningful change because the absorption process effected by *k_a__-fast_* would be ended in MTIME (=0.294 h) after drug administration. The *k_a__-slow_* was decreased since the absorption process could be prolonged for GI stimulation by GGT intake [26]. It meant the residence time of drugs from the stomach to the small intestine was increased, so when AAP was co-administered with GGT, the absorption rate constant of AAP could be smaller by a prolonged absorption process than AAP administered alone.

In our population PK result, GGT increased *V/F* while it had no effect on *CL/F* of AAP, and sequentially resulted in an increase in *t*_1/2_ of AAP. Our result about no effect on *CL/F* also confirmed with previous reports that GGT may have no effect on AAP hepatic metabolism. This result is likely consistent with the study of Lee et al. [27], in which the authors reported that GGT at 0–300 ug/mL had no effect on AAP metabolic enzymes (CYP3A4, CYP 1A2, CYP 2A6, CYP 2D6, CYP2E1). The increment of *V/F* by GGT could be explained by the fact that GE was increased by a prolonged absorption process. In addition, no effect on hepatic metabolism did not mean that no interactions in GI metabolism, the potential interaction between AAP and GGT in GI metabolism could be in GI metabolism. However, the limitation of knowledge of GGT or EPD on GI metabolism was we could not conclude about the potential interaction in GI. This would be promised future work to figure out exact mechanisms of interaction.

An increase in *t*_1/2_ will lead to an increase in accumulation of plasma level if accumulation occurs after multiple dose regimen. If the successive dosage is administered before the previous doses are completely eliminated, plasma concentration will be accumulated. A drug is considered completely eliminated (around 97% of a drug) from body after five half-life [28]. In our simulation scenario of AAP 4000 mg per day (1000 mg × 4 times), dosing interval (6-h) is lesser than five times of *t*_1/2_ of AAP in both G1 and G2, therefore accumulation occurs in both groups. The extent of drug accumulation is calculated as the following Equation (6) [29]:

AR = 1/(1 − *e*^-*k*×*tau*^) (6)

where AR is accumulation ratio, *k* is the terminal elimination rate constant, and *tau* is the dosing interval.

According to this equation, AR is 1.18 and 1.63 in AAP alone group and combined group (AAP + GGT), respectively. The extent of drug accumulation in AAP alone group is lower than that of combined group. It results in an increase in *AUC* of AAP in combined group comparing to that in AAP alone group at dose of 1000 mg × 4 times per day with an increase of approximately 116.4% of *AUC*_inf_. This indicates an enhancement of efficacy and a greater risk of side effects as well. If > 4000 mg/day, severe liver injury can result [30], possibly because of saturation of sulfation and glucuronidation pathway in the liver. This increases hepatic NAPQI production and depletes NAPQI detoxification capacity [21], and eventually results in AAP-induced hepatotoxicity. Therefore, when co-administered with GGT, adverse effects related to overdose of AAP could be induced possibly.

A total of 1000 mg × 4 times per day of AAP was applied in the simulation scenario because it is the highest recommended dose of AAP for treatment of the common cold [22,23]. As reference, patients can use 1000 mg AAP every 6 h, and should not take more than 4000 mg in 24 h. In addition, AAP was reported to follow linear pharmacokinetics for doses of less or equal to 18 mg/kg [31]. Therefore, it is appropriate to extrapolate PKs of AAP 1000 mg × 4 times per day from AAP 1000 mg.

In addition to investigating the effect of EPD on PKs of AAP, we also checked the impact of AAP on PKs of EPD, in which AAP as perpetrator and EPD as victim. The NCA results and plot of concentration vs. time profile of EPD with/without AAP was provided in Supplementary Materials Table S3 and Figure S1, respectively. NCA results showed that there was no statistically significant difference between two groups, (EPD alone) and (EPD + AAP) (*p* > 0.05). Therefore, AAP was concluded to have no effect on PKs of EPD.

Pseudoephedrine (P-EPD), a stereoisomer of EPD, is widely combined with AAP in a fixed dose of many commercial products for treatment common cold, headache, fever, etc. [32]. P-EPD and EPD show a relatively similar in physiochemical properties, such as same solubility (8.26 mg/mL for both) [33,34], similar lipophilicity (logP = 1.13 for EPD [35], 0.89 for P-EPD [36]), etc. As physiochemical properties mainly govern PK properties of drugs in the body [37], PKs of P-EPD are comparable to PKs of EPD. As EPD has effects on PKs of AAP, P-EPD could have an interaction with AAP. Therefore, caution should be necessary when taking a combination of P-EPD and AAP, especially in multiple dosage regimen.

This study had several limitations. Movement of AAP from the stomach to the small intestine is a determinant of its absorption. In our study, subjects were fasted to exclude any effect of food on AAP absorption. However, food is typically consumed when a medicine is taken and the impact of food on AAP absorption should be taken into consideration. Moore et al. [38] reported that food delays the absorption of AAP. In the fed condition, the *T*_max_ was 1.32-fold longer than in the fasted condition. In addition, the fed *C*_max_ was 58% of the fasted *C*_max_. Further work is needed to investigate the influence of food on the absorption of AAP co-administered with GGT.

## 5. Conclusions

This study is the first study to describe the herb–drug interaction between GGT and AAP. The developed model is adequately described the PKs of AAP in presence or absence of GGT. Co-administered with GGT decreased *k_a__-slow_* approximately 3.5 times and increased *V/F* and *k_a__-fast_* approximately 5 and 8 times, respectively, comparing to AAP administered alone. Simulation results suggested that in the presence of GGT, the change in PKs of AAP was is unlikely to be clinically significant at AAP 1000 mg, however, at high doses (≥4000 mg AAP per day) the interaction will become worse and caution should be taken. Further study should be carried out to investigate such interactions in diverse populations and different conditions (for example, fed condition), to broaden representation.

## Figures and Tables

**Table 1:** The content of major ingredients in Galgeuntang.

Major Ingredient	Amount in One Tablet
Puerarin	3.65 mg
Ephedrine	2.63 mg
Paeoniflorin	6.07 mg
Glycyrrhetic acid	1.23 mg
(6)-shogaol	-

**Table 2:** Non-compartmental analysis (NCA) result of pharmacokinetic (PK) parameters of AAP.

Parameter	G1 (AAP alone) (*n* = 12) (Mean ± SD)	G2 (AAP + GGT) (*n* = 11) (Mean ± SD)	*P*-Value
*AUC*_inf_ (ng h/mL)	48,126 ± 9530	44,054 ± 10,330	*p* < 0.001
*C*_max_ (ng/mL)	14,441 ± 2896	10,212 ± 2151	*p* < 0.001
^‡^*T*_max_ (h)	0.250 (0.250–2.00)	0.250 (0.250–2.00)	-
*t*_1/2_ (h)	2.23 ± 0.274	4.39 ± 1.01	*p* < 0.001
*CL/F* (L/h)	21.6 ± 4.37	23.8 ± 5.53	*p* > 0.05
*V/F* (L/h)	69.2 ± 17.0	147 ± 36.7	*p* < 0.001

*AUC*_inf_, area under the plasma concentration–time curve from 0 to infinity; *C*_max_, maximum observed plasma concentration; *T*_max_, time to reach maximum observed concentration; *t*_1/2_, plasma half-life associated with terminal slope of a semilogarithmic concentration–time curve; *CL*, clearance; *V*, volume of distribution; *F*, bioavailability; SD, standard deviation. ^‡^*T*_max_ was presented as median (range).

**Table 3:** Model development of AAP.

Model No.	Model Description	OFV	ΔOFV	Compare with
1	One-compartment, first-order elimination, first-order absorption	1493		
2	Two-compartment, first-order elimination, first-order absorption	1527	+34.6	1
3	One-compartment, first-order elimination + Gastric emptying pattern	1487	−5.73	1
4	One-compartment, first-order elimination + two-phase absorption (*k_a__-fast_* + *k_a__-slow_*)	1485	−7.94	1

ΔOFV, change in objective function value.

**Table 4:** Summary of covariate model building steps.

Model No.	Model Description	ΔOFV	DF	Compare with	Significance (*p*-Value)
1	Base model	-	-	-	-
2	Base model, EPD as covariate on *CL/F*	Fail			
3	Base model, EPD as covariate on *K_a__-slow_*	−11.9	1	1	*p* < 0.05
4	Base model, EPD as covariate on *K_a__-fast_*	Fail			
5	Base model, EPD as covariate on *V/F*	−16.6	1	1	*p* < 0.05
3	Base model, EPD as covariate on *K_a__-slow_*	−11.9	1	1	*p* < 0.05
6	Base model, EPD as covariate on *K_a__-slow_*, *V/F*	−6.87	2	3	*p* < 0.05
7	Base model, EPD as covariate on *K_a__-slow_*, *V/F*, *K_a__-fast_*	−18.2	1	6	*p* < 0.05

ΔOFV, change in objective function value; DF: degree of freedom; *K_a__-slow_*, slow absorption rate constant; *K_a__-fast_*, fast absorption rate constant.

**Table 5:** Parameter estimates of the final covariate model and bootstrap result for AAP.

Parameters	Description	Final Model Estimate	RSE (%)	Bootstrap Median (n = 1000) Median (95% CI)
*CL/F* (L/h)	Apparent clearance	22.5	5.60	22.4 (20.6–24.1)
*V/F* (L)	Apparent volume of distribution	16.3	57.3	16.1 (5.60–29.0)
*K_a__-fast_* (1/h)	Rapid absorption rate constant	0.970	53.4	0.950 (0.360–2.32)
*K_a__-slow_* (1/h)	Slow absorption rate constant	0.320	9.50	0.320 (0.300–0.340)
*MTIME* (h)	The time at which absorption rate change	0.294	18.1	0.289 (0.250–0.342)
Prop.RE	Proportional random error	0.196	2.70	0.187 (0.145–0.233)
COV*_V/F_*	EPD as covariate on *V/F*	13.3	75.0	12.7 (5.80–45.3)
COV*_Ka__-slow_*	EPD as covariate on *K_a__-slow_*	2.28	11.2	2.23 (1.82–2.87)
COV*_Ka__-fast_*	EPD as covariate on *K_a__-fast_*	21.7	95.9	19.8 (6.95–69.6)
Ω*_CL/F_* (%)	Inter-individual variability of *CL/F*	19.8	43.4	
Ω*_ka__-fast_* (%)	Inter-individual variability of *K_a__-fast_*	33.3	24.7	

COV*_V/F_*, coefficient of covariate on *V/F*; COV*_Ka__-slow_*, coefficient of covariate on *K_a__-slow_*; COV*_Ka__-fast_*, coefficient of covariate on *K_a__-fast_*; RSE, residual standard error; CI, confidence intervals.

**Table 6:** NCA results of simulated AAP in steady state.

Group	*C*_max_ (ng/mL) Median (5–95% Percentile)	*AUC*_t_ (ng h/mL) Median (5–95% Percentile)	*AUC*_inf_ (ng h/mL) Median (5–95% Percentile)	*t*_1/2_ (h) Median (5–95% Percentile)
AAP (1000mg once per day)	10,872 (6627–19,042)	* 43,606 (25,436–71,008)	43,628 (25,449–71,047)	2.17 (2.16–2.17)
AAP (1000mg once per day) + GGT (7680 mg once per day)	9474 (6118–13,114)	* 43,024 (24,144–70,517)	43,883 (24,340–73,246)	4.69 (3.93–5.43)
AAP (1000mg × 4 times per day)	13,679 (8132–21,658)	^‡^ 40,940 (23,735–66,138)	51,855 (28,504–88,671)	2.17 (2.12–2.35)
AAP (1000mg × 4 times per day) + GGT (7680 mg once per day)	13,149 (8634–18,323)	^‡^ 42,854 (25,266–67,489)	60,357 (61,955–110,028)	2.80 (2.24–3.67)

* *AUC*_t_ in here is AUC in 24 h, ^‡^ *AUC*_t_ in here is AUC in 6 h.

## Supplementary Materials

The following are available online at https://www.mdpi.com/1999-4923/12/12/1182/s1, Figure S1: Comparison of plasma concentration–time profiles of ephedrine after administration alone or co-administration with acetaminophen (AAP). Each point represents the mean and standard deviation. EPD, ephedrine, Figure S2: Representative chromatogram of blank plasma (A), blank plasma spiked with internal standard (B), standard sample of AAP at dose of 2 µg/mL (C), standard sample of EPD at dose of 0.5 µg/mL (D), real sample of AAP at 3 h after administration combination of AAP and Galgeuntang (GGT) (E), real sample of EPD at 3 h after administration combination of AAP and GGT (F), Table S1: Inclusion and exclusion of participants enrolling clinical trial, Table S2: Intra-day and inter-day of accuracies and precisions of AAP and EPD, Table S3: NCA results of PK parameters of EPD.

## References

[1] Williamson E.M. (2005). Interactions between herbal and conventional medicines. Expert Opin. Drug Saf..

[2] Yang T., Kim Y., Chae B. (2002). An experimental study on the anti-allergic effects, anti-inflammatory action, anti-pyretic action and analgesic action of Galgeun-tang, Gamigalgeun-tang and Geomahwanggalgeun-ang. J. Korean Orient. Med. Ophthalmol. Tolaryngol. Dermatol..

[3] Shin J.-M., Kim Y.-O., Baek S.-H. (2008). Free radical scavenging activity and kinetic behavior of the Galgeuntang water extract. Orient. Pharm. Exp. Med..

[4] Wu M.S., Yen H.R., Chang C.W., Peng T.Y., Hsieh C.F., Chen C.J., Lin T.Y., Horng J.T. (2011). Mechanism of action of the suppression of influenza virus replication by Ko-Ken Tang through inhibition of the phosphatidylinositol 3-kinase/Akt signaling pathway and viral RNP nuclear export. J. Ethnopharmacol..

[5] Ha H., Lee J.K., Lee M.-Y., Lim H.-S., Shin H. (2013). Galgeun-tang, an Herbal Formula, Ameliorates Atopic Dermatitis Responses in Dust Mite Extract-treated NC/Nga Mice. J. Korean Med..

[6] Son M.J., Kwon O., Kim S., Kim Y.E., Jung S.Y., Kim B.Y., Kang J.I., Lee J.H., Lee D.H. (2018). Safety and efficacy of Galgeun-tang-ga-cheongung-sinyi, a herbal formula, for the treatment of chronic rhinosinusitis: A study protocol for a randomized controlled trial. Medicine.

[7] Guerra M.C., Speroni E., Broccoli M., Cangini M., Pasini P., Minghetti A., Crespi-Perellino N., Mirasoli M., Cantelli-Forti G., Paolini M. (2000). Comparison between Chinese medical herb Pueraria lobata crude extract and its main isoflavone puerarin: Antioxidant properties and effects on rat liver CYP-catalysed drug metabolism. Life Sci..

[8] Wu W.H., Liu L., Han F.M., Chen Y. (2011). Effect of pseudoephedrine and ephedrine on the activities of cytochrome P450 enzymes in rat liver microsomes. China J. Tradit. Chin. Med. Pharm..

[9] Guo Y.J., Liang D.L., Xu Z.S., Ye Q. (2014). In vivo inhibitory effects of puerarin on selected rat cytochrome P450 isoenzymes. Pharmazie.

[10] Kim J.Y., Kim J.S., Jung J.H., Chun P., Rhew K.Y. (2014). Inhibitory effects of puerarin on cytochrome P450 subfamilies in vitro. Orient. Pharm. Exp. Med..

[11] Zhao K., Ding M., Cao H., Cao Z.X. (2012). In-vitro metabolism of glycyrrhetinic acid by human and rat liver microsomes and its interactions with six CYP substrates. J. Pharm. Pharmacol..

[12] Lv Q.-L.L., Wang G.-H.H., Chen S.-H.H., Hu L., Zhang X., Ying G., Qin C.-Z.Z., Zhou H.-H.H. (2015). In Vitro and in Vivo Inhibitory Effects of Glycyrrhetinic Acid in Mice and Human Cytochrome P450 3A4. Int. J. Environ. Res. Public Health.

[13] Hwang Y.P., Choi C.Y., Chung Y.C., Jeon S.S., Jeong H.G. (2007). Protective effects of Puerarin on carbon tetrachloride-induced hepatotoxicity. Arch. Pharm. Res..

[14] Zheng J., Chen B., Jiang B., Zeng L., Tang Z.R., Fan L., Zhou H.H. (2010). The effects of puerarin on CYP2D6 and CYP1A2 activities in vivo. Arch. Pharm. Res..

[15] Kim J. (2016). Effects of 6-Shogaol, A Major Component of Zingiber officinale Roscoe, on Human Cytochrome P450 Enzymes in vitro. Korean J. Med. Crop Sci..

[16] Jin S.E., Ha H., Jeong S.-J., Shin H.-K. (2014). Effects of Korean traditional herbal formula for common cold on the activities of human CYP450 isozymes. J. Korean Med..

[17] Li S., Li X., Yuan D., Wang B., Yang R., Zhang M., Li J., Zeng F. (2017). Effects of paeoniflorin on the activities and mRNA expression of rat CYP1A2, CYP2C11 and CYP3A1 enzymes in vivo. Xenobiotica.

[18] Kim S.B., Yoon I.S., Kim K.S., Cho S.J., Kim Y.S., Cho H.J., Chung S.J., Chong S., Kim D.D. (2014). In vitro and in vivo evaluation of the effect of puerarin on hepatic cytochrome P450-mediated drug metabolism. Planta Med..

[19] Li H.Y., Xu W., Su J., Zhang X., Hu L.W., Zhang W.D. (2010). In vitro and in vivo inhibitory effects of glycyrrhetinic acid on cytochrome P450 3A activity. Pharmacology.

[20] Bessems J.G., Vermeulen N.P. (2001). Paracetamol (acetaminophen)-induced toxicity: Molecular and biochemical mechanisms, analogues and protective approaches. Crit. Rev. Toxicol..

[21] Mazaleuskaya L.L., Sangkuhl K., Thorn C.F., Fitzgerald G.A., Altman R.B., Klein T.E., Alvarellos M.L., McDonagh E.M., Patel S., McLeod H.L. (2015). PharmGKB summary: Pathways of acetaminophen metabolism at the therapeutic versus toxic doses. Pharmacogenet. Genom..

[22] Uptodate.com Acetaminophen (Paracetamol): Drug Information. https://www.uptodate.com/contents/acetaminophen-paracetamol-drug-information.

[23] The Electronic Medicines Compendium Paracetamol 500 mg Tablets (P). https://www.medicines.org.uk/emc/product/9128/smpc#gref.

[24] Heading R.C., Nimmo J., Prescott L.F., Tothill P. (1973). The dependence of paracetamol absorption on the rate of gastric emptying. Br. J. Pharmacol..

[25] Clements J.A., Heading R.C., Nimmo W.S., Prescott L.F. (1978). Kinetics of acetaminophen absorption and gastric emptying in man. Clin. Pharmacol. Ther..

[26] Nimmo W., Heading R., Wilson J., Tothill P., Prescott L. (1975). Inhibition of gastric emptying and drug absorption by narcotic analgesics. Br. J. Clin. Pharmacol..

[27] Lee S.Y., Lee J.Y., Kang W., Kwon K.I., Park S.K., Oh S.J., Ma J.Y., Kim S.K. (2013). Cytochrome P450-mediated herb-drug interaction potential of Galgeun-tang. Food Chem. Toxicol..

[28] Nnane I.P. (2004). Pharmacokinetics—Absorption, Distribution, and Elimination. Encyclopedia of Analytical Science: Second Edition.

[29] Ahmad A.M. (2007). Potential pharmacokinetic interactions between antiretrovirals and medicinal plants used as complementary and African traditional medicines. Biopharm. Drug Dispos..

[30] Lee W.M. (2017). Acetaminophen (APAP) hepatotoxicity—Isn’t it time for APAP to go away?. J. Hepatol..

[31] Sahajwalla C.G., Ayres J.W. (1991). Multiple-dose acetaminophen pharmacokinetics. J. Pharm. Sci..

[32] Drugs.com Acetaminophen and Pseudoephedrine. https://www.drugs.com/mtm/acetaminophen-and-pseudoephedrine.html#:~:text=Acetaminophenandpseudoephedrineisa,listedinthismedicationguide.

[33] Drugbank.com Ephedrine. https://go.drugbank.com/drugs/DB01364.

[34] Drugbank.com Pseudoephedrine. https://go.drugbank.com/drugs/DB00852.

[35] PubChem Ephedrine. https://pubchem.ncbi.nlm.nih.gov/compound/9294#section=Vapor-Pressure.

[36] PubChem Pseudoephedrine. https://pubchem.ncbi.nlm.nih.gov/compound/Pseudoephedrine#section=Vapor-Pressure.

[37] Loftsson T. (2015). Physicochemical Properties and Pharmacokinetics. Essential Pharmacokinetics.

[38] Moore R.A., Derry S., Wiffen P.J., Straube S. (2015). Effects of food on pharmacokinetics of immediate release oral formulations of aspirin, dipyrone, paracetamol and NSAIDs—A systematic review. Br. J. Clin. Pharmacol..

