# Supplementary material for: Clinical Evaluation of Acetaminophen–Galgeuntang Interaction Based on Population Approaches
Source: Pharmaceutics. 2020 Dec 4;12(12):1182. doi: 10.3390/pharmaceutics12121182 (PMC7761965; doi:10.3390/pharmaceutics12121182)
Supplement: Supplementary file 1 [file pharmaceutics-12-01182-s001.pdf]

pharmaceutics

Supplementary Material: Clinical Evaluation of Acetaminophen-Galgeuntang Interaction Based on Population Approaches

Quyen Thi Tran, So Jung Park, Hyun-moon Back, Lien Thi Ngo, Duc Tuan Cao, Hung Van Nguyen, Sangkeun Jung, Jung-woo Chae, Yang Chun Park and Hwi-yeol Yun

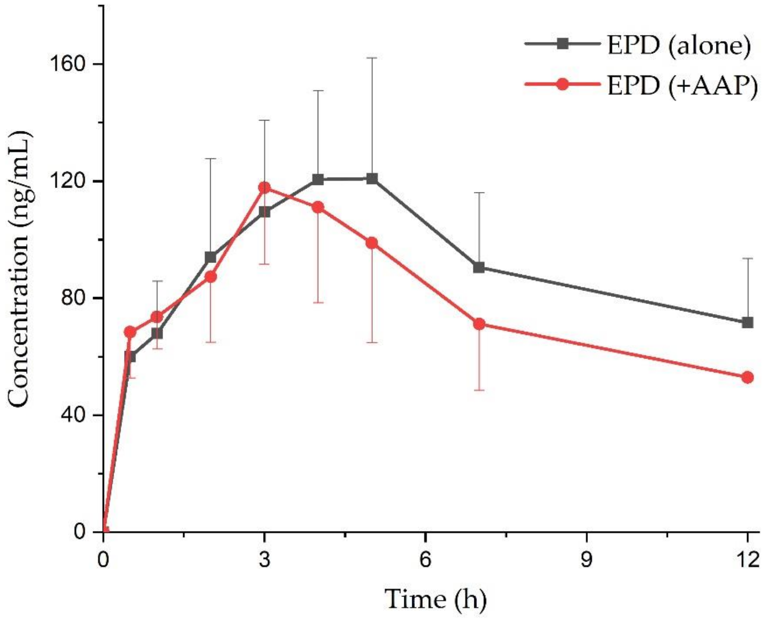

Figure S1. Comparison of plasma concentration-time profiles of ephedrine after administration alone or co-administration with acetaminophen (AAP). Each point represents the mean and standard deviation. EPD, ephedrine.

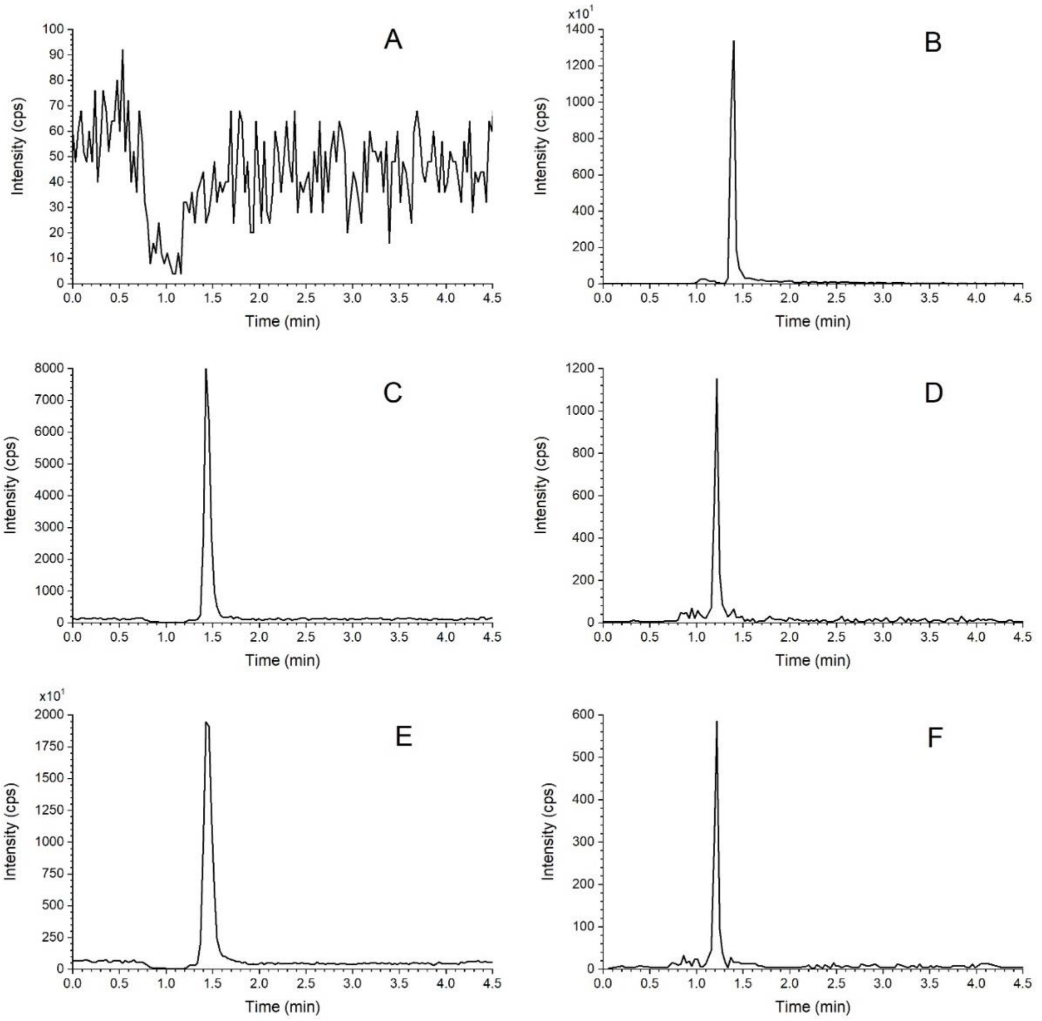

Figure S2. Representative chromatogram of blank plasma (**A**), blank plasma spiked with internal standard (**B**), standard sample of AAP at dose of 2 µg/mL (**C**), standard sample of EPD at dose of 0.5 µg/mL (**D**), real sample of AAP at 3 h after administration combination of AAP and Galgeuntang (GGT) (**E**), real sample of EPD at 3 h after administration combination of AAP and GGT (**F**).

Table S1. Inclusion and exclusion of participants enrolling clinical trial.

Criteria	Contents
Inclusion criteria	1. Men age from 20 to 45 years old<br>2. Body weight ≥55 kg, and within ±20% IBW [1] (ideal body weight)<br>3. Subject without any significant chronic disease<br>4. No pathological conditions at time of screening<br>5. Adhere the protocol and sign in an informed consent form
Exclusion criteria	1. Inflammatory fever<br>2. History of clinically significant hepatic, kidney, digestive system, pulmonary, blood, endocrine, urinary, neurology, musculoskeletal, immune, and cardiovascular systems<br>3. History of gastrointestinal surgery or gastrointestinal disorders which may importantly affect absorption of drugs<br>4. Aspirin-induced asthma<br>5. Clinically significant allergies, including GGT components or AAP<br>6. Clinically significant vital sign:<br>-Systolic blood pressure > 140 or < 100 mmHg<br>-Diastolic blood pressure > 90 or < 60 mmHg<br>-Heart rate ≥ 100 beats/min<br>7. Clinical laboratory test values are outside the accepted normal range:<br>-AST or ALT ≥ 2 times to the upper limit value<br>-Total bilirubin > 2 times the upper limit value<br>8. Use of prescription medicines within 14 days or non-prescription medicines within 7 days before dosing<br>9. History of drug abuse<br>10. Renal impairment (Creatinine clearance < 50 mL/min)<br>11. Using drugs which related to inhibition or induction of our drugs within 1 month before dosing<br>12. Participated in other study within 3 months before dosing<br>13. Bleeding tendency in blood test<br>14. History of alcohol, smoking abuse:<br>-Alcohol > 210g/weeks<br>-Smoking ≥ 10 cigarettes/day<br>15. Donated blood within 2 months; received or had blood transfusion within 1 month before dosing<br>16. Subjected considered as unsuitable based on medical judgement by investigators

Table S2. Intra-day and inter-day of accuracies and precisions of AAP and EPD.

Compounds	Concentration (ng/mL)	Intra-day		Inter-day	
		Precision (%RSD)	Accuracy (%RE)	Precision (%RSD)	Accuracy (%RE)
Acetaminophen	50	−1.06	6.92	3.94	4.49
	2000	−0.853	5.58	1.17	5.11
	10,000	−0.0405	2.54	−5.21	3.85
Ephedrine	50	8.99	6.44	4.65	3.44
	500	−2.79	3.50	−6.06	6.25
	2500	3.67	3.02	3.07	4.28

RSD: relative standard deviation, RE: relative error.

Table S3. NCA results of PK parameters of EPD.

Parameter	Mean ± SD		*p*-value
	EPD (single) *n* = 12	EPD (+AAP) *n* = 11	
$AUC_{0\text{-}t}$ (ng h/mL)	969 ± 249	833 ± 217	$p > 0.05$
AUC_{inf} (ng h/mL)	1399 ± 496	1206 ± 313	$p > 0.05$
C_{max} (ng/mL)	142 ± 32.6	126 ± 27.3	$p > 0.05$
T_{max} (h)	3.38 ± 1.11	3.55 ± 1.04	$p > 0.05$
$t_{1/2}$ (h)	5.82 ± 2.11	6.01 ± 3.22	$p > 0.05$
CL/F (mL/h)	25.1 ± 8.86	28.0 ± 8.14	$p > 0.05$
V/F (mL/h)	195 ± 60.7	225 ± 79.7	$p > 0.05$

AUC_{inf}, area under the plasma concentration-time curve from 0 to infinity; C_{max}, maximum observed plasma concentration; T_{max}, time to reach maximum observed concentration; $t_{1/2}$, plasma half-life associated with terminal slope of a semilogarithmic concentration-time curve; *CL*, clearance; *V*, volume of distribution; *F*, bioavailability; SD, standard deviation.

Reference:

1. Syahputra, M.F.; Felicia, V.; Rahmat, R.F.; Budiarto, R. Scheduling Diet for Diabetes Mellitus Patients using Genetic Algorithm. *J. Phys. Conf. Ser.* **2016**, *755*, doi:10.1088/1742-6596/755/1/011001.
